# Primary aortoduodenal fistula associated with abdominal aortic aneurysm with presentation of gastrointestinal bleeding: a case report

**DOI:** 10.1186/s12872-018-0852-y

**Published:** 2018-06-07

**Authors:** Tzu-Chieh Lin, Chung-Lin Tsai, Yao-Tien Chang, Sung-Yuan Hu

**Affiliations:** 1 Department of Emergency Medicine, Taichung Veterans General Hospital, No. 1650 Taiwan Boulevard Sect. 4, Taichung, 40705 Taiwan; 2 Divison of Cardiac Surgery, Cardiovascular Center, Taichung Veterans General Hospital, Taichung, Taiwan; 3 College of Public Health, China Medical University, Taichung, Taiwan; 4 Department of Nursing, College of Health, National Taichung University of Science and Technology, Taichung, Taiwan; 5 School of Medicine, Chung Shan Medical University, Taichung, Taiwan; 6 Institute of Medicine, Chung Shan Medical University, Taichung, Taiwan; 7 Department of Nursing, Central Taiwan University of Science and Technology, Taichung, Taiwan

**Keywords:** Abdominal aortic aneurysm (AAA), Aortoduodenal fistula (ADF), Computed tomographic angiography (CTA), Endovascular aortic repair (EVAR), Gastrointestinal (GI) bleeding

## Abstract

**Background:**

Primary aortoduodenal fistula (ADF) is a rare cause of gastrointestinal (GI) bleeding and is difficult to diagnose as the clinical presentation is subtle. Clinicians should keep a high level of suspicion for an unknown etiology of GI bleeding, especially in older patients with or without abdominal aortic aneurysm (AAA). Computed tomographic angiography (CTA) can be used to detect primary ADF. Open surgery or endovascular aortic repair (EVAR) for ADF with bleeding will improve the survival rate.

**Case presentation:**

We report a rare case of AAA complicating ADF with massive GI bleeding in a 73-year-old Taiwanese man. He presented with abdominal pain and tarry stool for 5 days and an initial upper GI endoscopy at a rural hospital showed gastric ulcer only, but hypotension with tachycardia and a drop in hemoglobin of 9 g/dl from 12 g/dl occurred the next day. He was referred to our hospital for EVAR and primary closure of fistula defect due to massive GI bleeding with shock from ADF caused by AAA. Diagnosis was made by CTA of aorta.

**Conclusions:**

A timely and accurate diagnosis of primary ADF may be challenging due to insidious episodes of GI bleeding, which are frequently under-diagnosed until the occurrence of massive hemorrhage. Clinical physicians should keep a high index of awareness for primary ADF, especially in elderly patients with unknown etiology of upper GI bleeding with or without a known AAA.

## Background

Aortoenteric fistula (AEF) is a rare but life-threatening condition with an annual incidence of 0.007 per million [1]. The clinical picture of AEF is characterized by a “herald GI bleeding”, followed by massive bleeding and exsanguination. The time interval between the herald bleeding and the exsanguination ranges from hours to months [2]. Most primary AEF is caused by an expanding native aorta with compression of an aortic aneurysm (AA) against the gastrointestinal (GI) tract. Atherosclerotic change is found in 85% of AEF-related AA [3]. Primary AEF is the cause of less than 0.2% of all GI bleeding and complicating only 0.7–2% of AA. Primary aortoduodenal fistula (ADF) is extremely rare with an incidence rate at autopsy of 0.04–0.07% and compromises 80% of all AEF cases [3–5]. Computed tomographic angiography (CTA) is a good diagnostic tool for ADF [6]. Endovascular aortic repair (EVAR) could be applied as a “bridge” treatment in hemodynamically unstable patients in specific situations [5].

## Case presentation

**Fig. 1:**
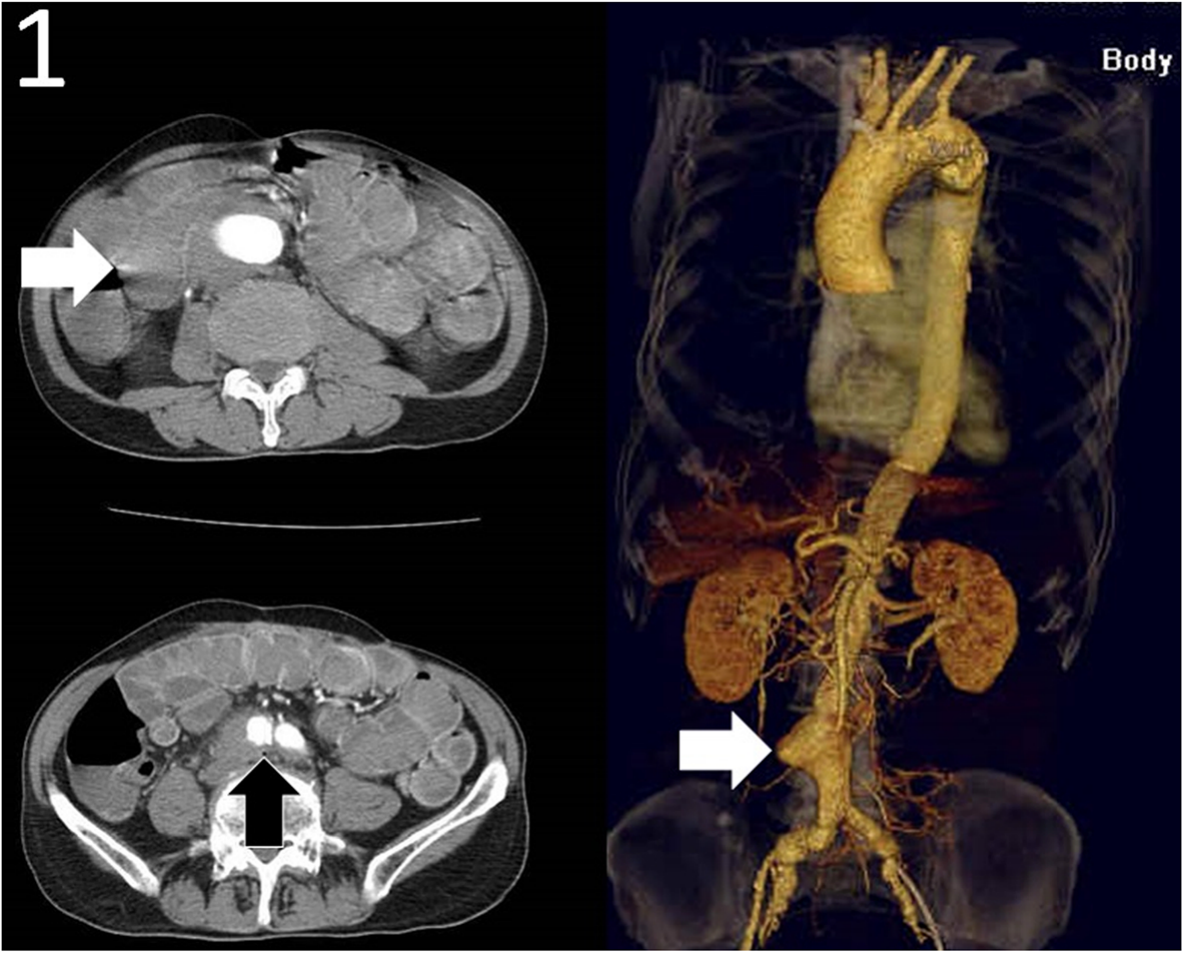
Computed tomographic angiography (CTA) of aorta depicted swollen and dilated bowel loop, contrast within bowel loop (white arrow), air bubble (black arrow), and an abdominal aortic aneurysm (AAA) (white arrow in reconstructed image of CTA)

A 73-year-old man had a past operative history of ruptured colonic diverticulitis treated by Hartmann’s procedure in 2007, and perforated peptic ulcer treated by pyloroplasty in 2009. He suffered from abdominal and tarry stool for 5 days in March 2011, so he was admitted to a rural hospital for further survey and medication. An initial upper GI endoscopy showed gastric ulcer only, but hypotension with tachycardia and a drop in hemoglobin of 9 g/dl from 12 g/dl occurred the next day. Intravenous fluid and blood transfusion with packed red blood cell were prescribed. Abdominal aortic aneurysm (AAA) with rupture was highly suspected, so CTA of aorta (Fig. 1) was carried out which disclosed an AAA with swollen bowel loop. He was transferred to our emergency department (ED) for consideration of vascular surgical intervention.

On arrival in our ED, vital signs were a respiratory rate of 16 breaths per min, a heart rate of 130 beats per min, a blood pressure of 98/73 mmHg, and a body temperature of 35.6 °C. Physical examination revealed a pale conjunctiva, no heart murmur, clear breathing sound, old operative scar over middle abdomen, and hypoactive bowel sound with tenderness over the periumbilical region. Leukocytosis, anemia, abnormal liver profiles, and metabolic acidosis were found. Laboratory investigations were summarized in Table 1.

**Table 1:** Summary of Laboratory investigations

Item (Unit)	Result	Reference range
White blood cell counts (/μl)	24,000	4000–11,000
Segments (%)	76.0	40–60
Hemoglobin (g/dl)	8.3	13.8–17.2
Platelet counts (× 10^3^/μl)	134	150–400
C-reactive protein (mg/dl)	7.25	< 0.4
Blood urea nitrogen (mg/dl)	14	10–25
Creatinine (mg/dl)	0.9	0.7–1.4
Sodium (mEq/l)	131	137–153
Potassium (mEq/l)	5.3	3.5–5.3
Calcium (mg/dl)	9.8	8.0–10.0
Blood glucose (mg/dl)	393	70–110
Albumin (g/dl)	2.0	3.5–5.0
Alkaline phosphatase (IU/l)	77	< 190
Aspartate transaminase (IU/l)	234	14–20
Alanine aminotransferase (IU/l)	166	10–40
Lactate dehydrogenase (IU/l)	685	< 240
Total bilirubin (mg/dl)	1.1	0.2–1.2
Amylase (IU/l)	371	40–140
Lipase (IU/l)	890	0–60
Arterial blood gas		
pH	7.328	7.35–7.45
P_a_O_2_ (mmHg)	241	80–100
P_a_CO_2_ (mmHg)	23.2	35–45
S_a_CO_2_ (%)	99.5	> 95
HCO3^−^ (mEq/l)	11.9	22–26

**Fig. 2:**
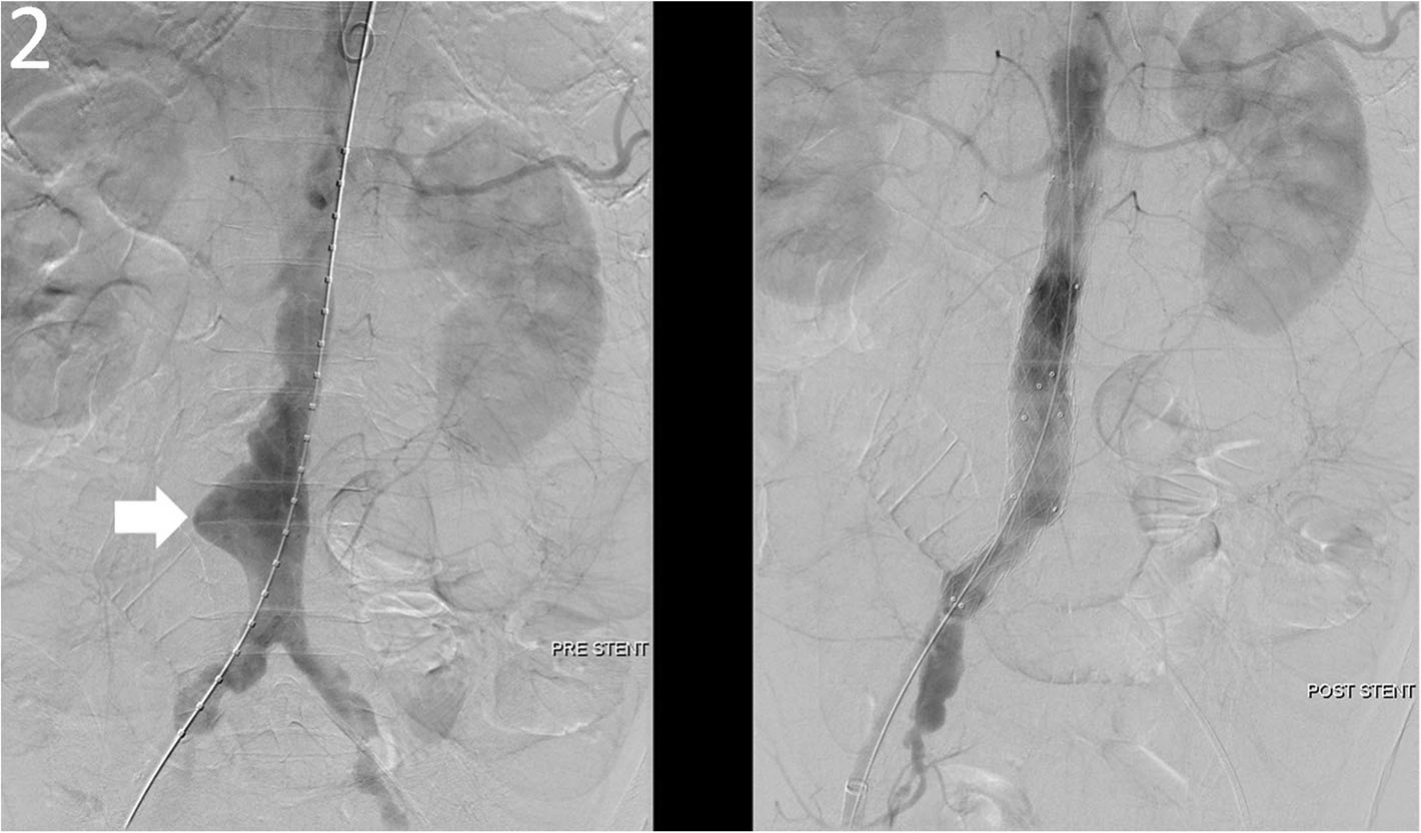
Angiography of aorta demonstrated an infrarenal AAA (white arrow in pre-stent) and a modified aorto-uni-iliac stent graft with resolution of an infrarenal AAA after implantation (post-stent)

**Fig. 3:**
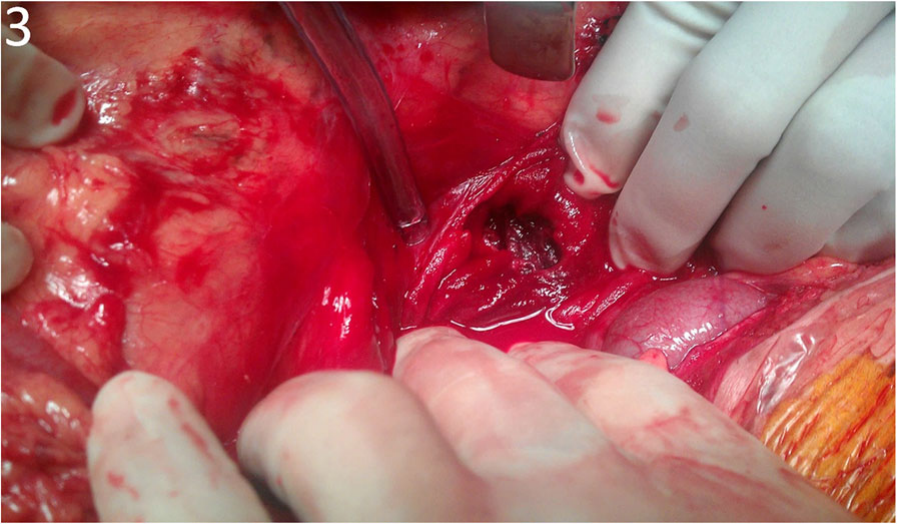
Exploratory laparotomy showed a fistula about 2x2cm over the third portion of duodenum

A cardiovascular surgeon carried out emergent angiography of aorta, which depicted bleeding from the wall of the AAA and chronic total occlusion of the left external iliac artery, so a modified aorto-uni-iliac stent graft (Fig. 2) was implanted with femoral-to-femoral bypass, which underwent smoothly. A general surgeon performed exploratory laparotomy and found a massive blood clot with a volume of about 1310 ml filling the entire lumen of the small intestine, a dilated and swollen duodenum about 6 cm in diameter with ecchymosis, and a fistula about 2x2cm over the third portion of the duodenum (Fig. 3). Duodenostomy with primary closure for repair of fistula defect, partition of the duodenum between the 2nd and 3rd portions, and side-to-side gastrojejunostomy were completed smoothly. He was admitted to the surgical intensive care unit for postoperative care. CTA of aorta showed neither endoleakage nor intraabdominal abscess on postoperative day 17. He was discharged without complications and returned for follow up at the out-of-patient department on postoperative day 25.

## Discussion and conclusions

Aortoenteric fistula (AEF), a direct communication between the aorta and the GI tract [1]. AEFs can be classified into primary and secondary types according to the presence or absence of a prior history of aortic surgery. Secondary AEFs, which occur in patients who have had previous aortic prosthetic reconstruction and are produced by an erosion of the aortic prosthesis into the GI tract, are 10 times more frequent than primary AEFs [7]. Primary AEFs are associated with spontaneous rupture of the expanding aorta into a closely adherent portion of the GI tract, which may lead to GI bleeding with presentation of a minor or intermittent hemorrhage (herald bleeding) in the initial stage or life-threatening exsanguination in the late stage [2]. Primary AEFs are clinically revealed by GI bleeding in approximately 80% of the cases. The GI bleeding is usually self-limited, with a secondary massive hemorrhage occurring within the next six hours in one-third of cases [3]. The clinical picture of AEF is characterized by a “herald GI bleeding”, followed by massive bleeding and exsanguination. The time interval between herald bleeding and exsanguination ranges from hours to months [3]. Most primary AEF involves the expansion of AAA with chronic mechanical compression of the GI tract, which leads to fibrotic change and inflammatory destruction. The most commonly involved digestive tract is the third and fourth portions of the duodenum, accounting for 80% of AEF cases [3, 5]. The classic triad of GI bleeding, abdominal pain, and pulsating abdominal mass, occurs in 25% of primary AEF cases [1, 4].

Primary aortoduodenal fistulas (ADF) is an abnormal communication between the infrarenal aorta and duodenum involving the 3rd part of the duodenum in two-thirds of cases and the 4th part in one-third of cases [6]. Primary ADF is an extremely rare clinical entity. There were 791 ADF cases of reported between 1951 and 2010, including 253 cases of primary ADF and 491 cases of secondary ADF [4, 8, 9].

CTA of aorta can reveal the size, location, and degree of calcification of an AAA, so it is a good diagnostic tool for ADF with a sensitivity of 40–90% and a specificity of 33–100%. Loss of the aneurysmal wall, air within the aortic wall, retroperitoneum or thrombus, focal bowel wall thickening with destruction of the fat plane between the aneurysm and duodenum, or contrast within the GI tract strongly suggest ADF in CTA findings [2, 3, 6]. However, aortography with evidence of contrast extravasation into the bowel was only positive in 26% of cases [2]. Upper GI endoscopy is the preferred primary modality for obtaining with valuable diagnostic information in a hemodynamically stable patient with GI bleeding, but it rarely reveals confirmatory evidence of a primary ADF (detection rate of 25%) because stable patients often do not have active bleeding [2, 4]. Diagnosis of primary ADF is also difficult. Only 33 to 50% of AEF are diagnosed preoperatively [5].

The therapeutic approaches for primary ADF are open surgery or endovascular repair [1]. If it is left untreated or misdiagnosed, the mortality rate is 100%. Endovascular aortic repair (EVAR) could be applied in specific situations as a “bridge” treatment in hemodynamically unstable patients to allow enteric repair with definitive intention, which would result in a better outcome when an open surgical procedure is either difficult or contraindicated [5]. Malnutrition increases post-surgical morbidity and mortality, hospital stance, and economical costs in patients of AEF. Continued nutritional support is essential in the postoperative course, but a feeding jejonostomy or gastrostomy is not recommended at least during the first five to seven post-operative days due to potential complications related to tube enterostomies. The use of parenteral nutrition is a method to support the nutritional status if the anticipated prolonged absence of oral food intake [10].

In conclusion, a timely and accurate diagnosis of primary ADF may be challenging due to insidious episodes of GI bleeding, which are frequently under-diagnosed until the occurrence of massive hemorrhage. Clinical physicians should maintain a high index of awareness for primary ADF, especially in elderly patients with unknown etiology of upper GI bleeding with or without a known abdominal AA.

## Abbreviations

AAA: Abdominal aortic aneurysm
ADF: Aortoduodenal fistula
CTA: Computed tomographic angiography
EVAR: Endovascular aortic repair
GI: Gastrointestinal
